# Transfusions in patients with leukaemia admitted to an intensive care unit

**DOI:** 10.1186/2197-425X-3-S1-A248

**Published:** 2015-10-01

**Authors:** M Madsen, L Russell, J Stensballe, J Bonde, A Perner

**Affiliations:** Rigshospitalet, Copenhagen University Hospital, Copenhagen, Denmark

## Intr

Mortality among leukemia patients admitted to the intensive care unit remains high [1] and bleeding complications are common in particular among thrombocytopenic patients [2]. Platelet levels measured in thrombocytopenic ICU patients reflect both the inherit and transfused platelets. ICU patients generally receive a high amount of transfusions [3] and hematological ICU patients are no exception.

## Objectives

Our aim was to describe the number and type of transfusions given to patients with haematological malignancies admitted to an intensive care unit. Furthermore, we wanted to test if there was an association between the number of platelet transfusions given in the first three days after arrival at the ICU and 30 day-mortality.

## Methods

This was a retrospective observational study with data from patients with ALL, AML and MDS admitted to the ICU at Rigshospitalet, Copenhagen University Hospital 2008-2012. Data were extracted from the electronic ICU patient charts, blood bank database and national registries. To test for an association between platelet transfusions and 30-day mortality, we used logistic regression adjusted by SAPS II.

## Results

Our cohort included 112 patients. 104 (93 %) received at least one blood product with a median of 20 blood products (IQR 7.5-52.5). Eighty-five (76%) patients received one or more platelet transfusions (median 12, IQR 6-37). During the first three days in the ICU, 58 patients received at least one platelet transfusion (concentrate from 4 donors) (median 5, IQR 3-8). No association between the number of platelet transfusions given during the first three days in the ICU and 30-day mortality was observed (HR 1.004, 95%CI 0.895-1.126).

## Conclusions

In this cohort of patients with haematological malignancies admitted to the intensive care unit the use of blood products was high. Platelet transfusion given in the first three days in the ICU was not associated with increased 30-day mortality.

**Figure 1:**
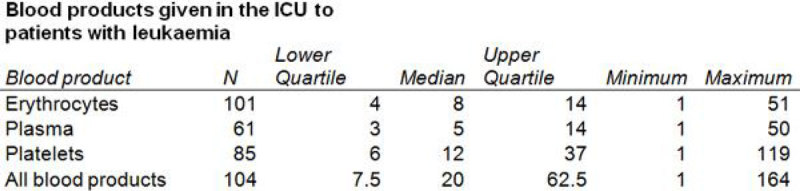
Figure 1
